# Anomaly Detection in the Production Process of Stamping Progressive Dies Using the Shape- and Size-Adaptive Descriptors

**DOI:** 10.3390/s23218904

**Published:** 2023-11-01

**Authors:** Liang Ma, Fanwu Meng

**Affiliations:** School of Mechanical Engineering, Beijing Institute of Technology, Beijing100081, China; liangma@bit.edu.cn

**Keywords:** stamping progressive die, anomaly detection, shape- and size-adaptive descriptor, machine vision

## Abstract

In the production process of progressive die stamping, anomaly detection is essential for ensuring the safety of expensive dies and the continuous stability of the production process. Early monitoring processes involve manually inspecting the quality of post-production products to infer whether there are anomalies in the production process, or using some sensors to monitor some state signals during the production process. However, the former is an extremely tedious and time-consuming task, and the latter cannot provide warnings before anomalies occur. Both methods can only detect anomalies after they have occurred, which usually means that damage to the die has already been caused. In this paper, we propose a machine-vision-based method for real-time anomaly detection in the production of progressive die stamping. This method can detect anomalies before they cause actual damage to the mold, thereby stopping the machine and protecting the mold and machine. In the proposed method, a whole continuous motion scene cycle is decomposed into a standard background template library, and the potential anomaly regions in the image to be detected are determined according to the difference from the background template library. Finally, the shape- and size-adaptive descriptors of these regions and corresponding reference regions are extracted and compared to determine the actual anomaly regions. The experimental results indicate that this method can achieve reasonable accuracy in the detection of anomalies in the production process of stamping progressive dies. The experimental results demonstrate that this method not only achieves satisfactory accuracy in anomaly detection during the production of progressive die stamping, but also attains competitive performance levels when compared with methods based on deep learning. Furthermore, it requires simpler preliminary preparations and does not necessitate the adoption of the deep learning paradigm.

## 1. Introduction

The progressive die is a stamping device that efficiently produces parts through continuous stamping of metal sheets, utilizing a press machine and a mold, based on the deformation theory of metal thin plates [1,2]. Smaller sheet metal parts that are needed in large quantities are typically manufactured using progressive dies due to the process’s stability, high production rate, and automation. Progressive dies have multiple stations, each performing one or more stamping operations [3]. The multi-station stamping progressive die is a type of advanced and efficient processing equipment for forming sheet metal parts, which can complete punching, bending, forming, and other stamping processes, and is widely used in modern industrial production. A characteristic of the production process of stamping progressive dies is periodic motion, which ensures highly stable production quality. However, the state of the progressive die often includes anomalies such as residual processing waste and foreign object splashes on the production line, as well as contamination and severe deformation of the workpiece [4,5]. If these anomalies occur at the stations of the progressive die and stamping continues, these anomalies may cause damage to the expensive mold and even pose a threat to production safety. Therefore, real-time monitoring of these stations and pausing the machine when anomalies occur at the stations are essential for protecting the mold and ensuring the normal operation of the processing process.

The early traditional approach involved workers constantly monitoring the products being manufactured on the production line. Upon detecting any anomalies in the products, the workers would immediately halt production and inspect the machinery. However, this was an extremely tedious and time-consuming process. Furthermore, the workers’ attention could potentially be diverted, leading to a delay in the detection of any abnormalities. To improve quality, state-of-the-art sensors are being used to replace visual inspections [6]. More recent methods for stamping process monitoring are based on the analysis of status signals derived from sensors installed on the processing equipment, which use the tonnage signature, acoustic signature, vibration signature, pressure signature, thermal signature, and other signatures as health indicators to determine the working condition of stamping progressive dies. Sah and Mahayotsanun et al. [7,8] used an array of tooling-integrated force sensors to measure contact pressure distribution across the sheet metal tooling interface for stamping process monitoring. Xu et al. [9] combined sensing techniques and the hidden Markov model to develop a fault diagnosis system, which enables adaptability and flexibility in monitoring industrial manufacturing processes. Li et al. [10] proposed an audio signal processing approach to inspect manufacturing equipment for tool wear. Kim et al. [11] integrated the principal component analysis technique and tonnage sensing system to perform stamping process inspection. These methods can identify an abnormal status in stamping equipment when malfunctions happen and, to some extent, avoid greater economic losses. Unfortunately, the biggest obstacle in the actual production process is the inability to monitor anomalies in real time, that is, to detect anomalies when they occur but have not yet caused a failure, and to immediately stop the operation of the machine. Currently, signal changes can only be detected when a failure has already occurred, which does not allow for taking measures in advance to limit the failure of processing equipment.

Machine vision technology can be employed for inspection purposes. Its fundamental principle involves the use of industrial cameras to continuously capture images of the target area, also known as the Region of Interest (ROI). Suitable algorithms are then applied to analyze the captured images to ascertain whether the target meets the requirements. Methods based on machine vision can achieve low-cost, high-precision, real-time inspection of target objects, without exerting any external influence on the production process [12]. Traditional computer vision techniques were often employed to detect surface defects in early studies. Ghorai et al. [13] employed wavelet features combined with a support vector machine to localize steel surface defects. Xie et al. [14] proposed an approach based on data augmentation and a support vector machine to detect defect patterns in noisy images. Liu et al. [15] proposed a model to project the local texture distribution into the low-dimension space, and an adaptive threshold was chosen to distinguish defects from the background. Truong and Kim [16] improved Otsu’s method via an entropy weighting scheme to segment small defect regions. Substantial research has been conducted on vision-based manufacturing process monitoring approaches. Martinez et al. [17] compared the information extracted from an industrial camera placed on top of a steel framing machine prototype with the manufacturing information available from the building information model to perform the pre-inspection of steel frame manufacturing. Lin [18] introduced a new adaptive vision-based method combining discrete wavelet transform-based feature extraction and support vector machine classification for automated inspection in manufacturing. Liu et al. [19] developed a product quality classifier based on a sparse multikernel least squares support vector machine to enable the supervision of assembly production lines. Gamage et al. [20] investigated possible defect detection methodologies and subsequently proposed a system capable of the real-time monitoring of defects in the cast extrusion manufacturing process.

So far, few vision-based methods have been proposed for online stamping process monitoring. The vision-based detection techniques related to stamping progressive dies mainly focus on offline workpiece quality monitoring, such as threshold-based methods [21,22], edge-based methods [23], and template matching-based methods [24], and these methods were compared in [25]. Stamping workpiece quality monitoring methods are mainly used for offline defect detection.

Although automatic surface defect detection via computer vision techniques has shown good performance in detecting specific surface defects, these methods could be further improved, since the complex feature extraction methods are often carefully designed based on the human experience.

In contrast, deep-learning-based automatic feature extraction methods have a strong pattern recognition ability, without requiring manual extraction of features. Researchers applied deep learning to surface defect detection, achieving greater accuracy than conventional methods. Networks such as VGG [26], GoogLeNet [27], and ResNet [28], which achieved high accuracy in natural image classification, have been applied to industrial images for classification [29,30,31,32] or feature extraction [33,34]. As a result, deep-learning-based defect detection methods have gained increasing popularity with applications in various industrial settings. Supervised methods are usually preferred when diversified and adequate defective samples can be easily collected and labeled. Yin [35] utilized Yolo V3 to detect damage defects in sewage pipelines and obtained 85.37% mean average precision (mAP). Feng [36] proposed an improved encoding–decoding network based on feature image fusion to detect cracks in hydroelectric dam images acquired by an unmanned aerial vehicle (UAV). Xiao [37] introduced a hierarchical-feature-based convolution neural network (H-CNN) model to detect oil leaks in freight trains. Due to the high level of standardization in industrial processes, instances of labeled damage patterns are seldom available. Infrequent deviations from normal conditions make it extremely challenging to gather an adequate number of labeled examples that accurately depict representative types of defects [38]. Manual delineation of the rectangular frame, as well as pixel-by-pixel segmentation, requires significant effort and assets, making collecting numerous defective samples and covering all defect types strenuous. A self-supervised learning strategy is capable of addressing these issues. Detone et al. [39] introduced a self-supervised framework for training interest point detectors that are applicable to multi-view geometry problems. In this framework, a homographic adaptation approach is proposed to generate pseudo-ground-truth interest points for self-supervised training. Araslanov and Roth [40] devised a data augmentation technique within a self-supervised framework that is trained on co-evolving pseudo labels, eliminating the need for cumbersome additional training rounds. Pautrat et al. [41] further expanded the self-supervised learning method in [39] to detect line segments. Xu [42] proposed SEDD, where a self-supervised learning strategy is utilized to address the scarcity of defective samples. Tasi et al. [43] proposed a reconstruction model based on convolutional autoencoders for the rapid and reliable detection of defects. These defects are trained using unsupervised learning strategies, which classify test images as defective or flawless but are unable to achieve pixel-level defect detection. Chow et al. [44] achieved good results in detecting concrete defects through the use of convolutional autoencoders to detect defects in concrete structures. Sean Givnan and colleagues applied autoencoders for anomaly detection in industrial motors [45]. Mishra et al. [46] proposed a novel transformer-based anomaly detection method that combines reconstruction-based methods with patch embedding. Wu et al. [47] proposed a self-supervised framework for comparison and recovery, which aims to learn generalized representations from unmarked defect images and improve the performance of various defect detection methods.

Dynamic motion scene abnormality monitoring methods are more suitable for stamping process monitoring. At present, dynamic scene modeling is used to detect moving objects in complex motion scenes. Common feature dynamic scene modeling methods include the hybrid Gaussian modeling algorithm, the Bayesian background modeling algorithm, the non-parametric kernel density estimation method, and the ViBe scenario modeling method [48,49,50]. However, the aforementioned modeling methods are not applicable when the entire Region of Interest (ROI) is in motion. This is because they all utilize the principle of re-siduals to subtract the pre-established background from the captured image in order to identify the moving regions. Consequently, they cannot detect deviations caused by loose parts in the device, nor can they protect the processing equipment with high accuracy.

In summary, the following issues exist in the monitoring process of the progressive die stamping production: (1) most equipment undergoes cyclical rather than static changes; (2) anomalies are sporadic and non-prior; (3) the workpiece will produce elastic deformation image differences; and (4) the pre-training of methods based on deep learning is time-consuming. To solve these problems, a shape- and size-adaptive descriptor (SSAD) is constructed, which is robust to all possible types of interference, to ensure high detection accuracy and thus ensure the normal operation of the machine.

The organization of this article is as follows. In Section 2, the methods applied to anomaly detection in the stamping process are described. Section 3 describes the comparative experiments conducted to verify the effectiveness of this method, where we compare our method with another descriptor and several popular deep-learning-based anomaly detection methods. The conclusions are described in Section 4.

## 2. Materials and Methods

Considering that the region of stamping production lines to be inspected is a periodic motion scene and the occurrence of anomalies is contingent and non-transcendental, a novel method for detecting anomalies in periodic scenes was developed in this study. The method consists of three main steps: (1) image segmentation, (2) SSAD construction, and (3) t-distribution-function-based anomaly region determination. This method was briefly introduced in our earlier work [51], and this paper will pro-vide a more detailed description of our approach.

### 2.1. Image Segmentation

The first stage of image segmentation involves building the standard template image library by decomposing the periodic motion scene of the stamping production line, using the method proposed by Wang et al. [52].

Assuming that the camera position and shooting angle are fixed, a continuous series of images of the station captured at continuous variable time *t*, $Im\left(x,y,t\right)$, is defined as its continuous motion scene. As suggested by Cutler and Davis [53], if the continuous motion scene of a station $Im\left(x,y,t\right)$ satisfies the equation

(1) $$Im\left(x,y,t\right)=Im\left(x,y,t+P\right),(P>0),$$

where *P* is a constant period, then the continuous motion scene is defined as a periodic motion scene.

To decompose the continuous motion scene into a discrete series of images, it is sampled with a constant sampling period *T*. If *T* satisfies the Shannon sampling theorem, the continuous motion scene information can be completely saved in a discrete series of images. The procedure for this can be mathematically described as multiplying $Im\left(x,y,t\right)$ by the sampling function $\delta_{T}\left(t\right)$ and then integrating the resulting product with respect to *t*:

(2) $$Im\left(x,y,nT\right)=\int_{0}^{+\infty }Im\left(x,y,t\right)\delta_{T}\left(t-nT\right)dt,\;\;\left(n=0,\pm 1,\pm 2\ldots \right).$$

When a stamping cycle is sampled, $\frac{P}{T}$ images of the stamping production line, $Im\left(x,y,nT\right)\;\left(\;n=1,2\ldots \frac{P}{T},\;and\;in\;the\;following,Im\left(x,y,nT\right)\;is\;marked\;as\;{Im}_{n}\right)$, are obtained. These images are defined as the standard template image library and can approximately replace $Im\left(x,y,t\right)$. Once the standard template image library has been constructed, the next step is to select the best matching image for the image to be detected, ${Im}_{wt}$, which is captured during production and is used to inspect the stamping production line for anomalies.

A clustering strategy is utilized to accelerate the matching step. It starts from the image with the smallest subscript picked among standard template images that do not yet fall within a cluster and then calculates the similarity measure, *Sim*, between this image and the image with the smallest subscript in the last cluster. *Sim* can be computed using the following formula:

(3) $$Sim=\frac{1}{1+\sum_{x=1}^{M}\sum_{y=1}^{N}\left|{Im}_{1}\left(x,y\right)-{Im}_{2}\left(x,y\right)\right|}$$

where ${Im}_{1}$ and ${Im}_{2}$ represent images, and *M* and *N* are the size of the image. If the similarity measure is larger than a given threshold, this image will be assigned to the last cluster. Otherwise, we build a new cluster with it. We proceed this way until every image belongs to a cluster. By first computing the similarity measure between the image ${Im}_{wt}$ and the images with the smallest subscript of every cluster, we can find the cluster most similar to ${Im}_{wt}$. We further calculate the similarity measure between ${Im}_{wt}$ and every standard template image in this cluster and define the image that causes the similarity measure to take the maxima as the best matching image of ${Im}_{wt}$.

**Figure 1:**
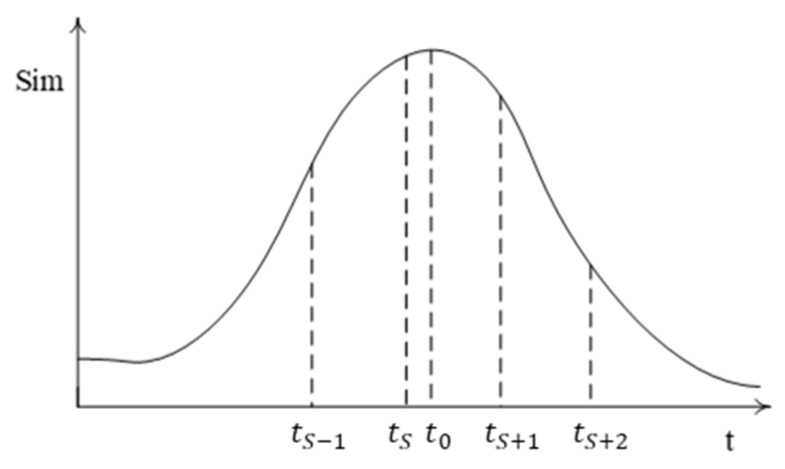
*Sim–t* curve.

The similarity curve of the periodic motion scene $Im\left(x,y,t\right)$ and the image ${Im}_{wt}$ is shown in Figure 1.

The curve takes the maximum value at $t_{0}$, and $t_{s}=t_{s-1}+T$, $t_{s+1}=t_{s}+T$. However, the template image corresponding to $t_{0}$ is often not available because the standard template image library is a discrete series of images. In practice, the image ${Im}_{s}$ corresponding to $t_{s}$ that is closest to $t_{o}$ is determined as the best matching image for ${Im}_{wt}$. A time interval $t_{g}$ $\left(0\leq t_{g}<T\right)$ exists between the image ${Im}_{wt}$ and standard template image ${Im}_{s}$, in which a certain translation occurs between the two images in the spatial domain.

The image pair is calculated by matching the image ${Im}_{wt}$ to the standard template image library. Thereafter, image registration is carried out to align this image pair. The geometric transformation model between the standard template image ${Im}_{s}$ and image ${Im}_{wt}$ can be approximated as the affine transformation model:

(4) $$\left(\begin{array}{c} u\\ v\\ 1 \end{array}\right)=\left(\begin{array}{ccc} a_{11} & a_{12} & t_{1}\\ a_{21} & a_{22} & t_{2}\\ 0 & 0 & 1 \end{array}\right)\left(\begin{array}{c} x\\ y\\ 1 \end{array}\right).$$

Considering that the feature-based image registration method offers relatively high accuracy and efficiency [54] and that SURF features exhibit certain robustness to noise and affine transformation [55,56], the features of images ${Im}_{s}$ and ${Im}_{wt}$ are extracted using the method developed by Bay et al. [55]. Thereafter, by means of feature matching, calculation of the affine transformation model parameters, image transformation, and resampling steps, the image pair is registered [57].

The registered image ${Im}_{rgs}$ is obtained by transforming the image ${Im}_{wt}$ into the standard template image ${Im}_{s}$. Then, the difference image ${Im}_{df}$ can be calculated using the following formula:

(5) $${Im}_{df}\left(x,y\right)=\left\{\begin{array}{c} 1,\;\;\;\left|{Im}_{rgs}\left(x,y\right)-{Im}_{s}\left(x,y\right)\right|\geq {Th}_{gr}\\ 0,\;\;\;\left|{Im}_{rgs}\left(x,y\right)-{Im}_{s}\left(x,y\right)\right|<{Th}_{gr} \end{array}\right.$$

where ${Th}_{gr}$ is the Otsu threshold. To enhance the robustness to high-frequency detail interference, a combination of spatial and morphological filtering is performed. (1) Prior to the threshold step, spatial filtering is conducted on $\left|{{Im}_{rgs}-Im}_{s}\right|$ with a Gaussian filter. The purpose is to filter out the high-frequency detail interferences contained in ${Im}_{df}$. (2) Once the difference image is obtained, we filter out the 8 small connected regions in ${Im}_{df}$ as they can be considered as interferences that step (1) failed to remove.

**Figure 2:**
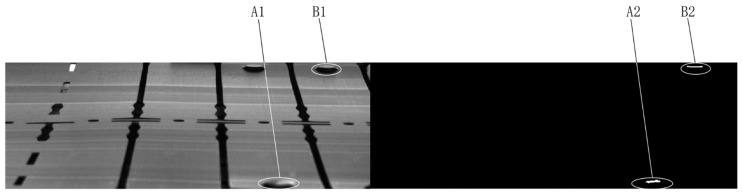
Example of a local bright spot region (**A1**), a bright-spot-caused interference region (**A2**), a local elastic deformation region (**B1**), and an elastic-deformation-caused interference region (**B2**).

**Figure 3:**
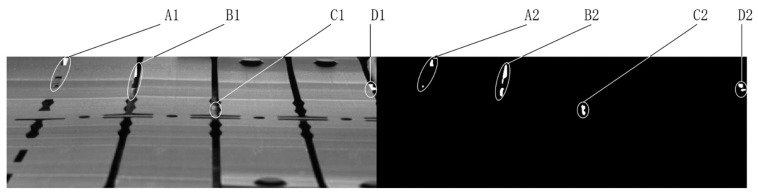
The hole structures in the workpiece (**A1**–**D1**) and corresponding interferences in the difference image (**A2**–**D2**).

The obtained difference image may contain connected regions representing anomalies and/or interferences. All possible types of interference regions and the cause of their presence in the difference image ${Im}_{df}$ are discussed in detail:

(a) As the actual production environment is versatile and complicated, the collected images often contain local bright spots owing to the partial reflection of the workpiece (see region A1 in Figure 2). These local bright spots also cause the generation of interference regions in the difference image (see region A2 in Figure 2).
(b) Local elastic deformation of the workpiece owing to external forces during production and inherent errors in image registration methods may lead to a complex local transformation between certain regions in ${Im}_{rgs}$ and their corresponding regions in ${Im}_{s}$, which may result in interference regions, such as region B2 in Figure 2. The transformation model between the region pairs is simplified as a translation model when the high-order distortion terms can be omitted.
(c) The background exposed through the hole structure on the workpiece may also result in interference regions in the difference image (see Figure 3). A background image of the workpiece is captured to remove such an interference region.

**Figure 4:**
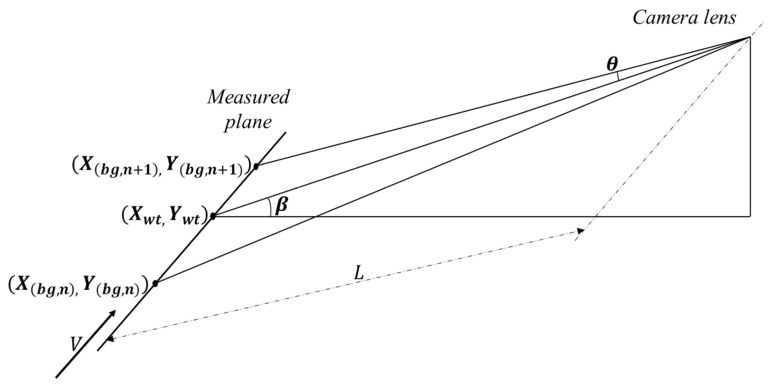
A sketch map of the theoretical maximum angle of view change, *θ*.

Due to the constraints of the real scene in the production of stamping progressive dies, it is difficult for the camera to shoot from directly above the stamping parts to achieve the highest resolution and reduce image distortion, thereby achieving the minimum information loss according to the Shannon sampling theorem. In practice, shooting can only occur from the side of the stamping parts. The geometric modeling [52] of the experimental scene is shown in Figure 4. It is assumed that the target's movement speed is *V*, and the direction of speed is shown in the figure. The camera shoots at a downward angle of β, degrees, and L is the distance from the lens to the target point. Therefore, in this model, the theoretical maximum viewing angle change θ in the image to be measured and the corresponding background library is calculated as shown in Formula (6).

(6) $$\theta =arc\;tan\frac{V\cdot T}{2L}$$

When the height of an object in the scene is h, then the maximum occlusion pixel pt caused by the perspective change due to side shooting along the direction of motion is calculated as shown in Formula 7.

(7) $$pt=\frac{h\cdot tan\theta }{sin\beta }\cdot r=\frac{hVT}{2Lsin\beta }\cdot r$$

In this model, pt represents the maximum error, and r denotes the image resolution. Given that β is small, sinβ can be approximated as 1, and since L is significantly larger than VT, the value of pt tends to be small. This implies that the error introduced by pt is within a tolerable range and can be further minimized through subsequent morpho-logical steps. Consequently, the equivalent diameter (in pixels) for anomaly detection is as follows:

(8) $$d=\sqrt{{\left(pt+e_{vib}+e_{reb}\right)}^{2}\cdot sin\beta }$$

In this context, *e_vib_* represents the error attributed to vibration, while *e_reb_* signifies the error resulting from registration. Utilizing this method can substantially streamline the scene and address the issue at hand.

### 2.2. SSAD Construction

As previously discussed, there are often interference regions in *Im_df_*, necessitating further investigation to ascertain whether the connected regions in the difference image represent actual anomalies. Based on the fact that there are two corresponding regions in ${Im}_{rgs}$ and ${Im}_{s}$ (candidate region and reference region) for a connected region in ${Im}_{df}$, we compare the descriptors describing the candidate regions with those describing the reference regions to complete this task. However, the existing feature descriptors are for image features, and they are of fixed shapes and sizes. If these descriptors are utilized directly to describe the characteristics of anomalies, information other than that of anomalies will be included. If the scale of the anomaly is small, its information that is reflected in the descriptor will be reduced relative to the total information contained in the descriptor. Therefore, descriptors of fixed sizes and shapes are less distinctive when describing the characteristics of anomaly regions.To overcome this issue, we propose a distinctive SSAD for the connected region. By calculating the matching distances between the SSADs of candidate and reference re-gions, we can identify connected regions that contain anomalies.

To construct the SSAD, we identified the left vertical tangent and top horizontal tangent of a connected region. A rectangle was then formed with the intersection point of the two tangents as a vertex. This rectangle, which could contain the connected region and sides in the vertical or horizontal direction, had a size that was an integer multiple of 3 s. Here, s was the sampling step, which depended on the area of the connected region *A* and was determined as follows:

(9) $$s=\left[\frac{A}{{4A}_{0}}\right]+1$$

**Figure 5:**
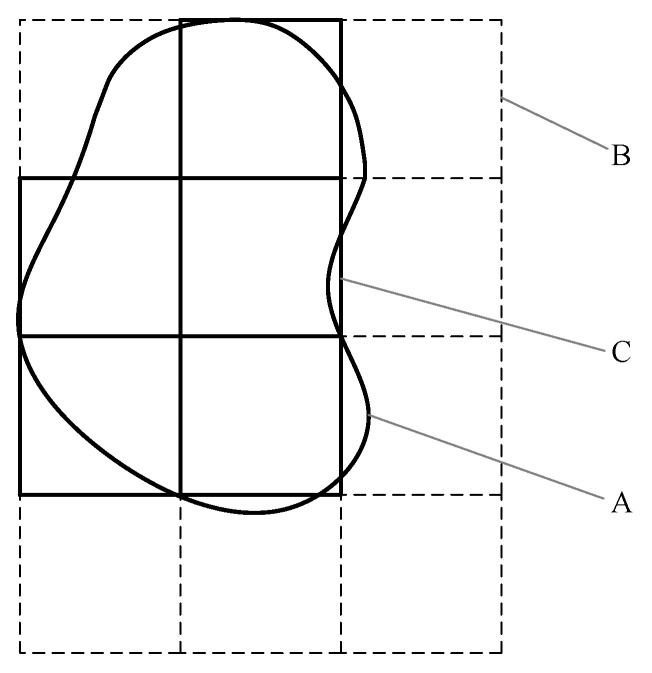
Sketched map depicting splitting of connected region into 3×3 square sub-regions: (**A**) connected region, (**B**) rectangle, and (**C**) remaining sub-region.

We propose a method where the rounding function is denoted as [], and we suggest a constant *A_0_*, typically set to 30 in this paper. We further partitioned the rectangle into square sub-regions of 3 s size in a regular manner. Sub-regions whose centers did not fall within the connected region were eliminated, as depicted in Figure 5. The sample points from the connected region were derived by identifying a grid of 3 × 3 sample points in each remaining sub-region. Given the existence of a corresponding connected region in *Im_df_* for both the candidate and reference regions, from which the SSADs were to be extracted, and the definite positional relationship among these connected regions, we computed the sample points for each connected region in *Im_df_*. This allowed us to obtain the sample points of their corresponding connected regions.

**Figure 6:**
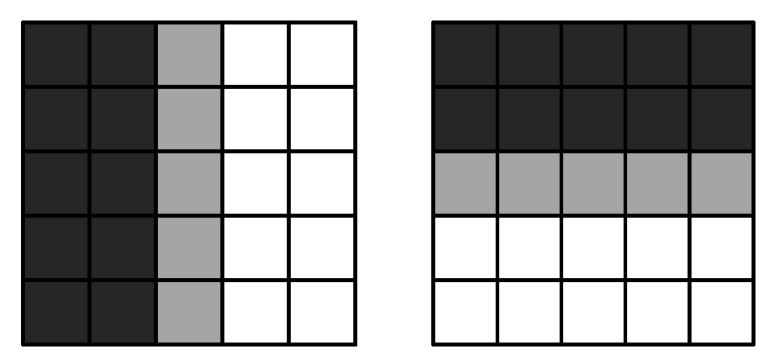
Haar wavelet filters for response dx (**left**) and response dy (**right**). The dark parts have a weight of −1, while the gray part is 0, and the light part is +1.

**Figure 7:**
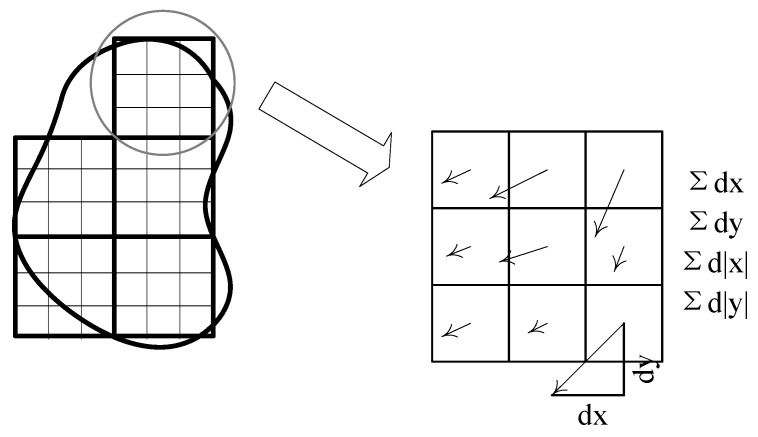
Sketch map of descriptor construction.

The above-mentioned procedures allocated sample points to the connected region. The next phase involved applying a Gaussian smoothing filter (*σ* = 0.01 × *A*) to the connected region, followed by determining the response at each sample point using the Haar wavelet filter, as shown in Figure 6. We denoted the operator response in the x-direction as dx and that in the y-direction as dy. Subsequently, we aggregated the responses *dx* and *dy* and their absolute values across each sub-region to yield $\sum dx,\sum \left|dx\right|,\sum dy,\sum \left|dy\right|$ , as depicted in Figure 7. For each sub-region, $\sum dx,\sum \left|dx\right|,\sum dy,\sum \left|dy\right|$ formed a 4D vector α.

(10) $$\alpha ={\left(\sum dx,\sum \left|dx\right|,\sum dy,\sum \left|dy\right|\right)}^{T}.$$

To counteract the influence of local bright spots, the 4D vector v was converted into a unit vector *e*

(11) $$e=\frac{\alpha }{\left|\left|\alpha \right|\right|}$$

The SSAD was computed by amalgamating the 4D vectors from all sub-regions of a connected region into an extended vector, β. If a connected region comprised n sub-regions, its SSAD, which was composed of the 4D vector extracted from each of its sub-regions, was a 4*n*D vector.

There are several reasons for choosing the aforementioned sampling steps for the connected regions of different areas and performing smoothing filtering when constructing the SSAD. Firstly, the cost of calculation of descriptor extraction can be decreased. Secondly, the lower resolution of the connected region of a larger area means it ignores certain noise details, and this increases the stability of the algorithm.

As mentioned previously, the error of the image registration and local elastic deformation may result in a spatial offset between two connected regions in ${Im}_{rgs}$ and ${Im}_{s}$ corresponding to a region on the workpiece. The translation of the reference region is undertaken to compensate for such an offset. The key to solving this problem is to identify the translation quantity. SURF uses the method proposed by Brown and Lowe [58] to determine the interpolated locations of the points of interest. Their approach uses the Taylor expansion of the scale-space function, $D\left(x,y,\sigma \right)$:

(12) $$D\left(X\right)=D+\frac{{\partial D}^{T}}{\partial X}X+\frac{1}{2}X^{T}\frac{\partial^{2}D}{{\partial X}^{2}}X,$$

where the origin of $D\left(x,y,\sigma \right)$ is at the sample point, and $X=\left(x,y,\sigma \right)$ is the offset from the origin. $\tilde{X}$ is obtained by calculating the derivative of *D* and setting it to zero, yielding

(13) $$\tilde{X}=-\frac{\partial^{2}D^{-1}}{\partial X^{2}}\frac{\partial D}{\partial X},$$

where $\tilde{X}$ is the offset from the sample point to the point of interest. If all elements of $\tilde{X}$ are greater than 0.5, the point of interest is near another sample point. The location of the point of interest is determined by adding the offset $\tilde{X}$ and the sample point location. According to Brown’s method [57], the theoretic error of the point-of-interest location is less than 0.5 in any dimension. The maximum registration error is obtained by substituting ${\left(0.5,0.5,0\right)}^{T}$ into Equation (4), yielding

(14) $${{\nabla X}_{rgs}=\left(0.5a_{11}+0.5a_{12},0.5a_{21}+0.5a_{22}\right)}^{T}.$$

The maximum reference region translation quantity, $X_{It}$, is obtained by substituting ${\nabla X}_{rgs}$ into the following equation:

(15) $$X_{It}=\frac{1}{\rho }X_{t}+{\nabla X}_{rgs},$$

where ρ is the realistic length represented by a pixel, and $X_{t}$ is the equivalent local translation quantity of the local elastic deformation quantity. The smallest step of the reference region translation is set to one. In particular, the translation quantities of the translated connected regions with respect to the original reference region can make up the set $S_{T}$:{$\left(u,v\right)|-x_{It}-1\leq u\leq x_{It}+1$, $-y_{It}-1\leq v\leq y_{It}+1,u,v\in Z$}, where ${\left(x_{It},y_{It}\right)}^{T}=X_{It}$. Following the reference region translation operation for the set of the connected regions, the SSAD of each connected region should be calculated. The key to this problem is to identify the sample points of each connected region. The coordinate of the sample point of the connected region X is expressed as the sum of the original reference region, $X_{r}$, and the translation quantity:

(16) $$X=\left(\begin{array}{c} u\\ v \end{array}\right)+X_{r}\;\left(u,v\right)\in S_{T}.$$

For a reference region, a set of SSADs (reference descriptor set) can be obtained after calculating the SSAD of each connected region produced thereby.

To remove the interference regions in ${Im}_{df}$ caused by the hole structures, for the connected region in ${Im}_{df}$, we compare the characteristics of the two corresponding regions in ${Im}_{rgs}$ and the background image. For this purpose, we calculate the SSAD of the corresponding region in the background image (background descriptor) of the connected region in ${Im}_{df}$. This involves the same steps as the construction of the SSAD of the candidate region and reference region. Note that the connected region in ${Im}_{df}$ and its corresponding region in the background image have different areas, shapes, and even positions. The coordinate of the sample point of its corresponding regions, $\left(u,v\right)$, can be obtained by substituting the coordinate of the connected region point in ${Im}_{df}$, $\left(x,y\right)$, into the following formula:

(17) $$\left(\begin{array}{c} u\\ v\\ 1 \end{array}\right)=T^{-1}\left(\begin{array}{c} x\\ y\\ 1 \end{array}\right),$$

where ***T*** is the transformation matrix from ${Im}_{wt}$ to ${Im}_{s}$.

### 2.3. t-Distribution-Function-Based Anomaly Region Determination

The previous operation constructed one candidate descriptor, one reference descriptor set, and one background descriptor for a connected region in ${Im}_{df}$. The next step is to infer the formula for anomaly region determination. The notation $\beta_{i}^{can}$ can be utilized to represent the candidate descriptor corresponding to the *i*’th connected region in ${Im}_{df}$ and $\beta_{ij}^{rob}$, the *j*’th reference descriptor corresponding to the *i*’th connected region in ${Im}_{df}$, or the corresponding background descriptor (*i =* 1, 2, …, *I*, and *j* = 1, 2, …, *J* + 1; *I* is the number of connected regions in ${Im}_{df}$, and *J* is the number of reference descriptors corresponding to a connected region in ${Im}_{df}$). In particular, $\beta_{ij_{0}}^{rob}$ corresponding to index $j_{0}$ represents the reference descriptor describing the connected region with a translation quantity of 0.

Since the majority of offsets between two connected regions in ${Im}_{rgs}$ and ${Im}_{s}$ corresponding to a region of the workpiece are negligible, let random variable $Y_{ij_{0}k}$ be the matching distance between the *k*’th 4D vector of $\beta_{i}^{can}$ and its corresponding 4D vector of $\beta_{ij_{0}}^{rob}$. According to the law of large numbers, $Y_{ij_{0}k}$ obeys normal distribution:

(18) $$Y_{ij_{0}k}\;\sim \;N\left(\mu ,\sigma^{2}\right),$$

where ~ represents “obey”. Let $\bar{Y}=\frac{1}{n}\sum_{i=1}^{I}\sum_{k=1}^{K_{i}}Y_{ij_{0}k}$, and $S=\sqrt{\frac{1}{n-1}{\sum_{i=1}^{I}\sum_{k=1}^{K_{i}}(Y_{ij_{0}k}-\bar{Y})}^{2}}.$ Then, Formula (19) is obtained by theoretical derivation:

(19) $$\frac{\bar{Y}-\mu }{\frac{S}{\sqrt{n}}}=\frac{\frac{\bar{Y}-\mu }{\frac{\sigma }{\sqrt{n}}}}{\sqrt{\frac{S^{2}\cdot \left(n-1\right)}{\sigma^{2}\cdot \left(n-1\right)}}}\sim t\left(n-1\right),$$

where $t\left(n-1\right)$ means t-distribution with a degree of freedom of *n* − 1; thus,

(20) $$P\left(\left|\frac{\bar{Y}-\mu }{\frac{S}{\sqrt{n}}}\right|<t_{\frac{\alpha }{2}}\left(n-1\right)\right)=1-\alpha .$$

By substituting the sample value of $Y_{ij_{0}k}$, $y_{ij_{0}k}$, and the sample value of $\bar{Y}$, $\bar{y}$, into Equation (20), Equation (21) is obtained:

(21) $$\mu <\frac{1}{\sqrt{n}}\sqrt{\frac{1}{n-1}{\sum_{i=1}^{I}\sum_{k=1}^{K_{i}}(y_{ij_{0}k}-\bar{y})}^{2}}\cdot t_{\frac{\alpha }{2}}\left(n-1\right)+\bar{y},$$

where *n* is the total number of 4D vectors in the image to be detected; $K_{i}$ is the number of 4D vectors in $\beta_{i}^{can}$; α is a confidence coefficient; and $t_{\frac{\alpha }{2}}\left(n-1\right)$ is the t-distribution upper $\frac{\alpha }{2}$ fractile.

Use the average matching distance over a connected region in ${Im}_{df}$ to approximately replace μ in Formula (21), and the formula for anomaly region determination is obtained. Given that the descriptor vector of the connected region is comprised of the 4D vectors of its sub-regions at varying positions, and these sub-regions hold different levels of significance, these 4D vectors are weighted using a Gaussian value ( σ = 1.5 s) at the center of the connected regions during the computation of the average matching dis-tance:

(22) $$E_{ij}=\frac{1}{h}\sum_{k=1}^{K_{i}}y_{ijk}\cdot \exp\left[-\frac{1}{2}{\left(X_{ij}-X_{i}^{cent}\right)}^{T}\Lambda^{-1}\left(X_{ij}-X_{i}^{cent}\right)\right],$$

where $X_{ij}$ is the center coordinate of the *j*’th sub-region of the *i*’th connected region in ${Im}_{df}$; $X_{i}^{cent}$ is the center coordinate of the *i*’th connected region in ${Im}_{df}$; $\Lambda =\left(\begin{array}{cc} \sigma^{2} & 0\\ 0 & \sigma^{2} \end{array}\right)$ is a diagonal matrix; $y_{ijk}$ is the sample value of the matching distance of the *k*’th 4D vectors, respectively, in $\beta_{i}^{can}$ and $\beta_{ij}^{rob}$; $E_{ij}$ is the average matching distance over the *i*’th connected region in ${Im}_{df}$ that is calculated from $\beta_{i}^{can}$ and $\beta_{ij}^{rob}$. Lastly, *h* is a normalization factor. This factor is introduced to counteract the influence of the Gaussian weight and standardize the matching distances of SSADs of varying sizes. The calculation of *h* is as follows:

(23) $$h=\sum_{k=1}^{K_{i}}\exp\left[-\frac{1}{2}{\left(X_{ij}-X_{i}^{cent}\right)}^{T}\Lambda^{-1}\left(X_{ij}-X_{i}^{cent}\right)\right].$$

For a connected region in ${Im}_{df}$, we obtain a set of average matching distances, $E_{i,1},E_{i,2}\ldots E_{iJ},E_{i,J+1}$. Let $E_{ij}^{min}=\min\left\{E_{i,1},E_{i,2}\ldots E_{iJ},E_{i,J+1}\right\}$. According to theoretical derivation, if the i’th connected region in ${Im}_{df}$ does not represent an anomaly region, $E_{ij}^{min}$ should satisfy the following equation:

(24) $$E_{ij}^{min}<\frac{1}{\sqrt{n}}\sqrt{\frac{1}{n-1}{\sum_{i=1}^{I}\sum_{k=1}^{K_{i}}(y_{ij_{0}k}-\bar{y})}^{2}}\cdot t_{\frac{\alpha }{2}}\left(n-1\right)+\bar{y}$$

Equation (24) is a theoretical formula. In order to increase its robustness and filter out interference, it should be further optimized. For that reason, another important parameter, known as the translation change rate (TCR), is introduced. The TCR ${cr}_{i}$ is obtained using the following formula:

(25) $${cr}_{i}=\frac{E_{i,j_{1}}+E_{i,j_{2}}}{\frac{2}{J+1}\sum_{j=1}^{J+1}E_{ij}}$$

where $E_{i,j_{1}}$ and $E_{i,j_{2}}$ are the maximum average matching distance and the second maximum average matching distance, respectively. The matching distance of the actual anomaly is almost constant when the translation quantity changes. Consequently, an actual anomaly has a small cr value. The matching distance of the normal region has the minimum value on the optimal translation quantity and increases sharply when the translation quantity deviates from the optimal translation quantity; thus, the normal region has a large cr value. This characteristic can be utilized to optimize Formula (24) by adding self-suppression.

In Formula (24), a few $y_{ij_{0}k}$ values come from the anomaly region. In order to reduce their impact on the accuracy of Formula (24), $y_{ij_{0}k}$ values from normal regions should be given a larger weight than those from anomaly regions. For this purpose, Formula (24) can be inferred:

(26) $$\frac{1}{\sqrt{n}}\sqrt{\frac{1}{n-1}{\sum_{i=1}^{I}\sum_{k=1}^{K_{i}}(y_{ij_{0}k}-\bar{y})}^{2}}\cdot t_{\frac{\alpha }{2}}\left(n-1\right)+\bar{y}=\sqrt{\frac{1}{n-1}\left(\bar{y^{2}}-{\bar{y}}^{2}\right)}{\cdot t}_{\frac{\alpha }{2}}\left(n-1\right)+\bar{y}$$

where $\bar{y^{2}}$ = $\frac{1}{n}\sum_{i=1}^{I}\sum_{k=1}^{K_{i}}{Y_{ij_{0}k}}^{2}$. At the same time, two weight coefficients are defined:

(27) $${Wight}_{i}^{1}=\frac{{cr}_{i}}{\sum_{i=1}^{I}{cr}_{i}}$$

and

(28) $${Wight}_{i}^{2}=\frac{{\left({cr}_{i}\right)}^{2}}{\sum_{i=1}^{I}{\left({cr}_{i}\right)}^{2}}.$$

$\bar{y}$ and $\bar{y^{2}}$ in Equation (26) are optimized as

(29) $$\bar{z}=\frac{1}{n}\sum_{i=1}^{I}\sum_{k=1}^{K_{i}}Y_{ij_{0}k}{Wight}_{i}^{1}$$

and

(30) $$\bar{z^{2}}=\frac{1}{n}\sum_{i=1}^{I}\sum_{k=1}^{K_{i}}{Y_{ij_{0}k}}^{2}{Wight}_{i}^{2}$$

respectively. By using t-distribution and self-suppression optimization, the interference regions’ impact is eliminated while determining the actual anomaly, which doubly enhances the robustness and accuracy of the method for anomaly detection.

Finally, through combining Equations (24), (26), (29), and (30), the formula for anomaly determination is obtained:

(31) $$E_{ij}^{min}<\sqrt{\frac{1}{n-1}\left(\bar{z^{2}}-{\bar{z}}^{2}\right)}{\cdot t}_{\frac{\alpha }{2}}\left(n-1\right)+\bar{z},$$

That is, if a connected region in ${Im}_{df}$ does not satisfy Formula (31), it is an anomaly region.

## 3. Comparative Experiments

In this section, we validate the advantages of our proposed method in the monitoring of progressive die stamping production through comparative experiments. Firstly, the experimental implementation is introduced in Section 3.1. In Section 3.2, we compare our proposed descriptor with a widely used descriptor, demonstrating the advantages of our method among non-learning methods. In Section 3.3, we compare our method with several popular deep-learning-based anomaly detection methods. The experimental results show that our method has competitive advantages compared to deep-learning-based methods.

### 3.1. Implementation of Proposed Method

The 800 T multi-station progressive die, a sophisticated and productive stamping die capable of executing stamping, bending, drawing, forming, and turning within a single die set, was utilized as the test subject to validate the aforementioned algorithm. This die can effectively produce a variety of complex parts. The ROI of the 800 T multistation progressive die production line can be considered a periodic motion scene. The manufacturing process is often plagued by anomalies such as foreign body splash-es (processed scraps, spitballs, stains) and machine part loosening, leading to damaged workpieces, waste products, and even equipment damage and malfunction, resulting in substantial economic losses. As a result, monitoring for anomalies in processing equipment has emerged as a crucial strategy for maintaining normal operations.

**Figure 8:**
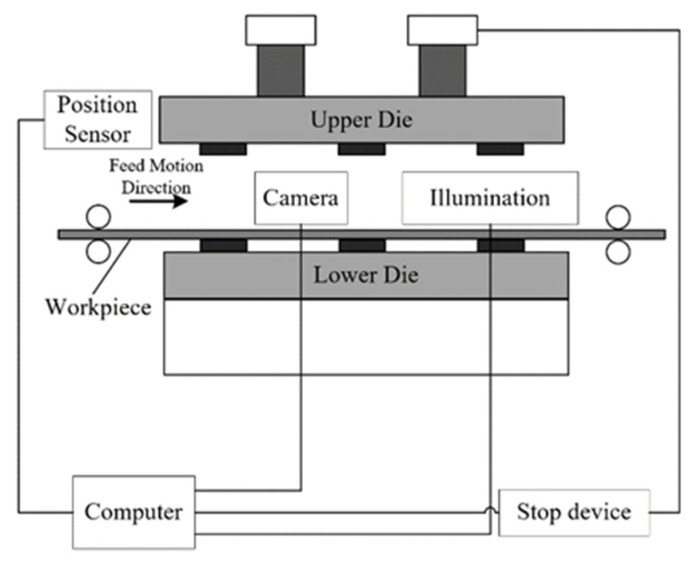
Configuration of detection system on 800 T multi-station progressive die.

The hardware of the detection system consists of a CMOS camera, a computer (Windows system), a planar light source, an auxiliary control system, and corresponding support equipment (see Figure 8). The core program of the detection system runs on the MATLAB 9.6.0 system. The camera is capable of producing 1.3 megapixel 60 fps grayscale images. The camera lens has a focal length of 25 mm, and the working distance L in this experiment was approximately 1.8 m. Four stations were chosen for detection from the collected imag-es, with a detection area of 851 × 371 pixels and a field of view of approximately 0.65 × 0.8 m^2^. Due to the scene‘s space constraints, the camera’s tilt angle β is relatively small, with β approximately equal to 21° in this experiment. In this scenario, the geometric relationship between pixels and reality on the X-axis and Y-axis is shown in Equation (32):

X-axis: 650 mm/851 pixel ≈ 0.76 mm/pixel Y-axis: sin(21°) × 800 mm/371 pixel ≈ 0.77 mm/pixel(32).

At this focal length, the corresponding relationship between the pixels of the focal image and the actual geometric size is ~0.8 mm per pixel. At this specific focal length, the pixel-to-actual geometric size correspondence in the focal image is approximately 0.8 mm per pixel. The camera is capable of capturing 30 images per second, with an interval of roughly 35 ms between each image and an ex-posure time of 3 ms. The detection environment, being a closed space, exhibits high re-silience to changes in illumination.

**Figure 9:**
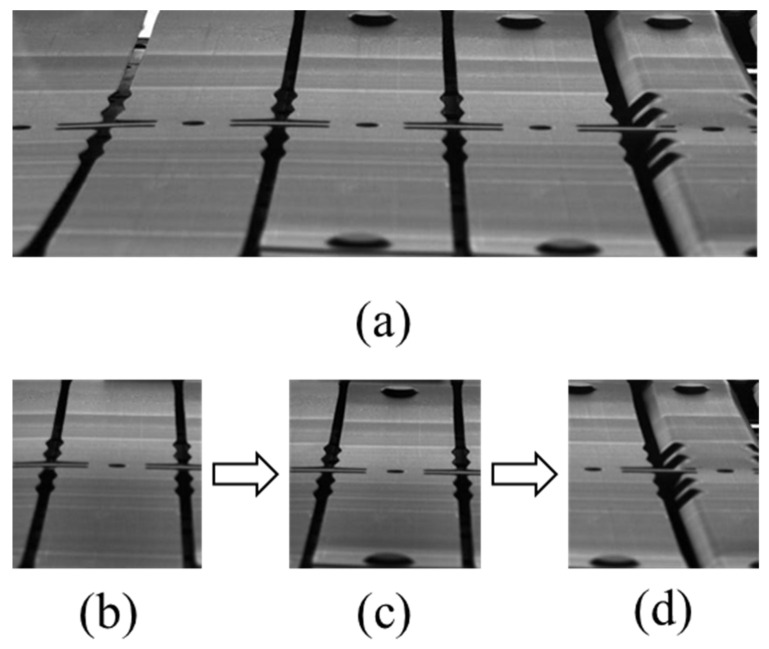
Progressive die stamping production process. (**a**) displays a part of the entire sheet metal processing process (**b**) is the workpiece to be punched, (**c**) is the state after punching, and (**d**) is the bending state.

The sheet metal workpiece to be processed is shown in Figure 9. Figure 9a displays a part of the entire sheet metal processing process, where three states appear simultaneously in Figure 9a: Figure 9b is the workpiece to be punched, Figure 9c is the state after punching, and Figure 9d is the bending state. The sheet metal workpiece moves to the right as a whole during processing, and the overall processing process is cyclical.

**Figure 10:**
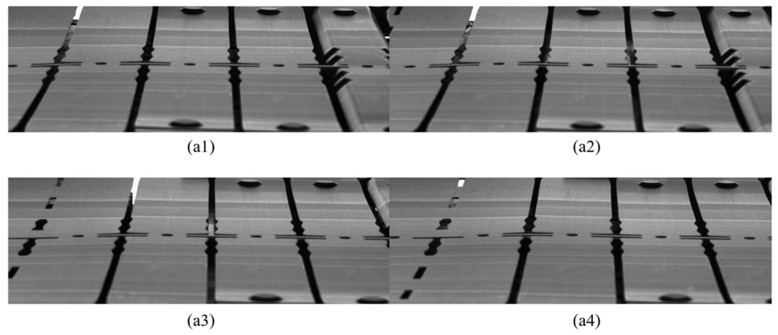
Images in standard template image library with corresponding numbers (**a1**) 7, (**a2**) 14, (**a3**) 21, and (**a4**) 28.

As discussed in Section 2, the construction of the standard template image library should precede the detection work. Image acquisition and detection are only possible when the die is open, limiting the detection to the time frame when a detectable image can be captured. The multi-station progressive die has an operating cycle of 3.5 s, and the effective image acquisition time is 1.4 s. Due to the fact that the detected area is completely stationary at the beginning and end of this cycle, and the camera exposure time is 3 ms, blur caused by vibration during shooting can be omitted. During this time, 30 images are captured with equal time intervals, then pre-processed, and finally saved as a standard template image library. Figure 10 illustrates part of the standard template image library.

### 3.2. Comparative Experiments with SURF

The SURF algorithm, short for Speeded-Up Robust Features, is an improvement over the SIFT operator. While it retains the excellent performance characteristics of SIFT, it addresses the high computational complexity and long computation time associated with SIFT. The SURF algorithm enhances the extraction of interest points and their feature vector descriptions, thereby speeding up computation. The specific steps of the SURF algorithm include constructing the Hessian matrix and calculating the eigenvalue α, building a Gaussian pyramid, locating feature points, determining the main direction of feature points, and constructing the feature descriptor. These steps ensure that SURF maintains the robustness of SIFT while significantly improving computational efficiency.

**Figure 11:**
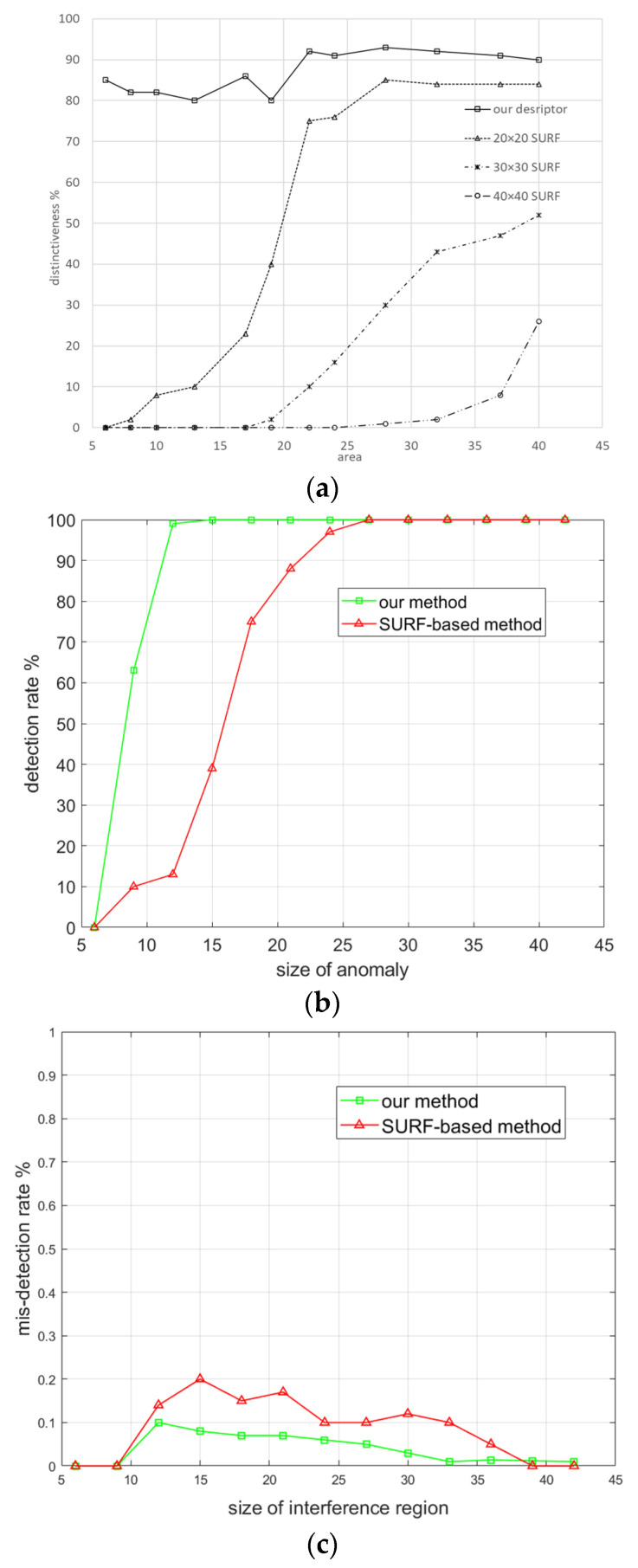
Comparison of methods applied on the die image set. Distinctiveness scores when increasing the sizes of foreign objects (**a**). Our descriptor’s score is greater than 80% for different sizes. Detection rate and misdetection rate for foreign objects of different sizes (**b**,**c**). Our method outperforms the SURF-based method when the foreign objects are relatively small.

In the experiments, our descriptor is compared to the SURF descriptors of different sizes, and performance evaluation is carried out on the die image set. Due to the lack of abnormal samples, we manually added external objects of different sizes to the progressive die and then took photos of the stamped parts with the foreign objects on them, to evaluate the distinctiveness of these descriptors. Anomalous sources may introduce foreign objects through various means, including but not limited to, the dispersion of extraneous materials, the spillage of waste, the de-tachment of components, the inclusion of pollutants in raw materials, and significant plastic deformation of the raw materials. Throughout the stamping process, the com-ponents consistently exhibited minor deformations. The distinctiveness scores of the different descriptors are shown in Figure 11a. This score is the ratio of the average matching distance of the anomaly region to ten times the average matching distance of the normal region. We also carried out experiments for detection rate and misdetection rate evaluation on the die image set, comparing our method to the SURF-based method presented in [51]. The results are illustrated in Figure 11b,c.

**Figure 12:**
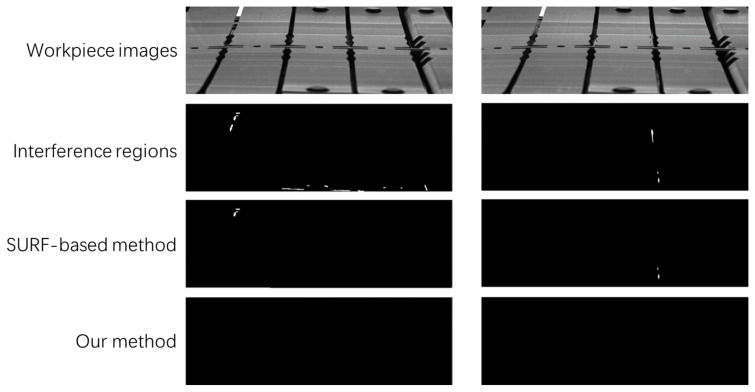
Comparison of our method with SURF.

Our method clearly outperforms the SURF-based method in detecting anomalies when the size of anomalies is less than 20 pixels. These very good performances can be explained by the fact that the SSAD contains less additional information and has a higher distinctiveness score. The SURF-based method has a relatively high misdetection rate. This can be traced back to its subpar performance in eliminating hole interferences, as illustrated in Figure 12. The figure presents four rows: the first row displays the work-piece under inspection, the second row shows the differential image with the interfer-ence area, the third row depicts the differential image post interference area filtering using the SURF-based method, and the fourth row presents the image after interfer-ence area filtering using our method. It is evident that the SURF-based method strug-gles with filtering out interference outside the hole.

The error check rate arises from the theoretical error introduced using the rigid body translation model to approximate the local elastic deformation and by using the affine transformation model to approximate the perspective transformation model. Furthermore, the discrete integer translation step is utilized to compensate for the registration error and elastic deformation, which introduces a discretization error. This type of error is a different source of the error check rate. The detection accuracy of this algorithm is affected by factors such as image resolution, camera tilt angle, and focal length. The resolution of the image is fixed and depends on the camera and lens selection. Because of factors such as registration error (about 1 pixel), machine vibration error (up to about 3 to 5 pixels), and the inherent error of the method (less than 1 pixel according to Equation (7)), according to Equation (8), the equivalent diameters detected under these conditions are

(33) $$\sqrt{{\left(1+1+4\right)}^{2}\cdot \sin21{}^{\circ}}\approx 3.6\;pixel\approx 3\;mm$$

As mentioned previously, five images were collected for testing in one cycle, and the detection of these five images should be completed before the next cycle to avoid production accidents caused by anomalies, whereby the effective detection time was 1.4 s. Therefore, the detection time of each image should be controlled within 280 ms. The algorithm had a detection time of 32 ms for an image without any optimization. The computer had an Intel i7-10700 CPU processor (Intel, Oregon, USA)with a clock speed of 2.9 GHz, 32 GB of RAM, and a 64-bit operating system. The core program ran under MATLAB 9.6.0, and the image resolution was 851 × 371 pixels; therefore, real-time detection was realized.

### 3.3. Comparison with the Methods Based on Deep Learning

We established a dataset that includes 500 images without anomalies and 10 images with anomalies, where the pixel positions of the anomalies have been manually annotated. We applied random rotation, cropping, and flipping to augment the data. After data augmentation, the number of images without anomalies reached 4000. The dataset also includes 500 images with anomalies and manually annotated pixel-level labels for training.

For training, we employed a batch size of 32 for training and an Adam optimizer with an initial learning rate of 0.0005. The cosine annealing strategy was utilized to adjust the learning rate, with the penalty parameter β set to 0.1. 

The proposed approach was implemented using Pytorch 1.7.0 and executed on a computer equipped with 32 GB of RAM, an Intel i7-10700 2.9 GHz CPU, an NVIDIA RTX 3080 GPU, and an Ubuntu 20.04 operating system.

We trained four standard segmentation and defect detection methods, namely SegNet [59], U-Net++ [60], MobileNetV2+DeepLabV3 [61], and PGA Net [62]. After the training was completed, the four learning-based methods were compared with our method on the same dataset. The test dataset consists of a total of 2000 images, of which 1987 are without anomalies, and 13 are anomalous. We used accuracy, miss rate, and time consumption to measure various methods. Accuracy refers to the proportion of correctly identified images to the total number of images, miss rate refers to images that were judged as normal but were actually abnormal, and time consumption refers to the time required to process a single image. The comparison results are shown in Table 1.

From the results, it can be seen that our method has achieved a high detection accuracy rate compared to several deep-learning-based methods, and our method has a miss rate of 0. In terms of time, all methods can achieve real-time monitoring in the stamping progressive die production process. However, the difference is that our method only needs 30 images in the image library to achieve extremely high accuracy monitoring.

**Figure 13:**
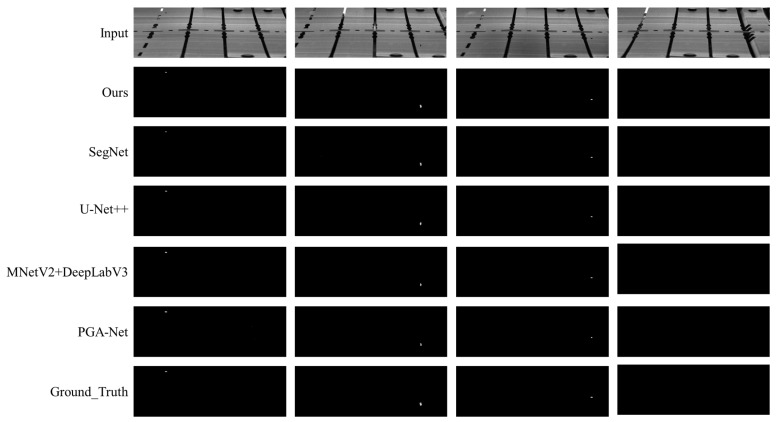
Visualization of the comparison of various methods.

Figure 13 a visualization of the comparison of various methods. Our method identifies anomalies through differential filtering with the proposed descriptor, while other methods do so through semantic segmentation. The first, second, and third columns display three types of anomalies, i.e., different foreign objects appearing on the workstation. The fourth column displays normal images.

Therefore, the monitoring method we proposed exhibits superior performance in scenarios with strong periodic characteristics such as stamping progressive die production, compared to deep-feature methods based on neural networks. This is because it requires a small image library that is easy to prepare and does not need to undergo the data processing and training processes required by deep learning paradigms. Moreover, it meets the demands of production in terms of accuracy and real-time performance.

## 4. Conclusions

This paper proposes a method for detecting anomalies in periodic motion scenes, which can be widely applied to production lines with these types of scenes. The proposed method has the following characteristics:

(1) The proposed SSAD for region description breaks the inherent mode of the traditional descriptor. Its adaptability to the shape and size of the anomaly region makes sure it is more distinctive. In constructing the SSAD, adaptive resolutions are used to describe the anomaly region, which reduces the computational cost of the feature extraction calculation, ignores high-frequency noise interferences, and improves the signal-to-noise ratio of the descriptor.
(2) This study introduced a novel method based on t-distribution for anomaly detection, which abandoned the traditional empirical theoretical threshold, showing a higher robustness. Meanwhile, self-suppression optimization based on TCR was used in this study, which drastically reduced the misdetection rate.
(3) The maximum translation quantity was inferred to filter out local elastic deformation, and the background descriptor was constructed to eradicate the impacts of backgrounds exposed through holes, reducing the misdetection miss rate to 0.0%.
(4) The proposed method outperforms the deep-feature method as it necessitates only a minimal number of images to construct an image library, and the level of detection can achieve results comparable to those of prevalent neural networks. At the same time, our method does not require paradigms such as knowledge transfer, pre-training, and fine-tuning of neural networks, making the preprocessing process simpler.

In this study, we used a progressive die to test this method. The experimental results show that the proposed algorithm can achieve comparable or even superior performance in terms of the anomaly detection rate compared to its counterparts and is superior in terms of the misdetection rate. Generally, processing equipment with periodic motion scenes, such as dynamic injection molds and printing machines, can be monitored using this method: first, a standard template image library is constructed during the periodic motion process of the mold, then the descriptor is constructed, and finally an anomaly region is determined based on the T-distribution function.

The focus of future work should be on conducting online monitoring tests of the method we proposed in the production of various processing equipment with periodic motion scenes. This will be the work of the next stage.

## Figures and Tables

**Table 1:** Comparison of test results using different methods.

Method	Number of Images Judged as Normal		Number of Images Judged as Abnormal		Accuracy (%)	Miss Rate (%)	Time Consumption (%)
	TRUE	FALSE	TRUE	FALSE			
Ours	13	3	1984	0	99.85	0.00	32
SegNet	10	4	1983	3	99.65	0.15	57
U-Net++	13	5	1982	0	99.75	0.00	37
MobileNetV2 +DeepLabV3	11	4	1985	0	99.8	0.00	33
PGA Net	11	3	1980	6	99.55	0.30	52

## Data Availability

Not applicable.
